# Qi-fu-yin attenuated cognitive disorders in 5xFAD mice of Alzheimer's disease animal model by regulating immunity

**DOI:** 10.3389/fneur.2023.1183764

**Published:** 2023-06-27

**Authors:** Xiuzhao Yang, Tianyuan Ye, Yun He, Lei Wen, Xiaorui Cheng

**Affiliations:** ^1^Innovative Institute of Chinese Medicine and Pharmacy, Shandong University of Traditional Chinese Medicine, Jinan, China; ^2^Xiamen Key Laboratory for TCM Dampness Disease, Neurology and Immunology Research, Department of Traditional Chinese Medicine, Xiang'an Hospital, School of Medicine, Xiamen University, Xiamen, Fujian, China

**Keywords:** Qi-fu-yin, Alzheimer's disease, learning and memory, cytokine, 5xFAD mice

## Abstract

**Introduction:**

Cognitive impairment is the main symptom of Alzheimer's disease (AD). Accumulating evidence implicate that immunity plays an important role in AD. Here, we investigated the effect of Qi-fu-yin (QFY) on cognitive impairment and cytokine secretion of 5xFAD mice.

**Methods:**

We used 2.5-month-old 5xFAD transgenic mice for behavioral tests to observe the changes in cognitive function after QFY treatment. After the behavioral experiment, the whole brain, cortex and plasma of each mouse were collected for soluble Aβ analysis, immunohistochemical experiment and cytokine analysis.

**Results:**

Here we found that the treatment of QFY ameliorated the ability of object recognition, passive avoidance responses and the ability of spatial learning and memory in 5xFAD mice. The deposits of β_1 − 42_ and Aβ_1 − 40_ were alleviated and the ration of Aβ_1 − 42_/Aβ_1 − 40_ was decrease in the plasma and brain of 5xFAD mice administrated with QFY. The administration of QFY promoted the secretion of anti-inflammatory cytokines, IL-5, IL-10 and G-CSF, and reduced the content of proinflammatory cytokines IFN-γ in plasma of 5xFAD mice. Notably, we found that the treatment of QFY decreased the concentration of CCL11 in the brain and plasma of 5xFAD mice.

**Conclusion:**

This suggested that QFY improved cognition and reduced Aβ deposits in 5xFAD mice by regulating abnormal immunity in 5xFAD mice. QFY may be as a potential therapeutic agent for AD.

## Introduction

Alzheimer's disease (AD) is an age-related degenerative disease of the central nervous system caused by multiple factors, accounting for ~60–80% of the incidence of dementia (1). AD is one of the most common diseases in the elderly, and its clinical manifestations are mainly characterized by cognitive deficit, language impairment, visuospatial impairment, slow thinking, inattention, emotional disorders, and personality changes, accompanied by the decline of social activities and self-living ability (2, 3). Pathologically, a large number of senile plaques were formed by Aβ protein aggregation, neurofibrillary tangles were formed by hyperphospho-tau protein, and a large number of neuron losses occurred in the brain of AD patients (4). The pathogenesis of AD is complex, and it is not clear yet. There is no ideal therapeutic drug in a clinic for AD.

Through the genetic research on the families of patients with AD, the researchers found three main pathogenic genes, namely, β-amyloid precursor protein (APP) gene, presenilin 1 (PS1) gene, and presenilin 2 (PS2) gene, which laid the foundation for the formation of Aβ to play an important role in the pathogenesis of AD (5). Microglia are activated by Aβ and then release cytokines, chemokines, and other neurotoxic substances (such as NO and superoxide), which lead to neuroinflammation in the brain of AD patients (6). The 5xFAD mice express gene APP with mutation K670N/M671L (Swedish), I716V (Florida), and V717I (London) and PS1 with mutation M146L and L286V. The 5xFAD mice develop stable and progressive plaque pathology, as well as synaptic dysfunction and neuron loss (7). In addition, the 5xFAD mice exhibit age-dependent learning and memory deficits (8, 9).

Traditional Chinese medicine prescription (TCMP) has the characteristics of multi-component, multi-target, and multi-pathway treating diseases. The Qi-fu-yin decoction (QFY), a TCMP, is composed of *Ginseng Radix Et Rhizoma, Rehmanniae Radix Praeparata, Angelicae Sinensis Radix, Atractylodis Macrocephalae Rhizoma, Glycyrrhizae Radix ET Rhizoma Praeparata Cum Melle, Ziziphi Spinosae Semen*, and *Polygalae Radix*. Modern clinical studies show that QFY can effectively treat AD (10, 11). Wang (12) searched multiple databases of clinical randomized controlled studies on QFY, and through a meta-analysis found that the clinical total effective rate, MMSE score, and HDS score of the experimental group were significantly higher than those of the control group. Experimental pharmacological research shows that QFY reduced escape latency of AGE-induced AD model rats in the Morris water maze test, decreased the levels of TNF-α, IL-1β, and AGE in the hippocampus, and downregulated the expressions of RAGE and NF-κB in the hippocampus and cortex of rats (13, 14). QFY increased the latency of the step-down test in Aβ-induced AD model rats and alleviated hippocampal neuron loss (15). Our previous research also found that QFY improved the aging of APP/PS1 transgenic mice by regulating the intestinal microbiome (16). However, there are few studies on QFY. The efficacy and mechanism of QFY treating AD need to be further confirmed and revealed.

In order to confirm the effects of QFY on AD, we used 5xFAD mice as a model to observe the effects of long-term administration of QFY on the ability of learning and memory and Aβ deposition and studied the regulatory effects of QFY on the levels of cytokines. We found that QFY improved the ability of learning and memory 5xFAD. In addition, QFY decreased the contents of plasma and cortex Aβ and regulated the levels of IL-17, IFN-γ, IL-5, IL-10, G-CSF, and CCL11.

## Materials and methods

### Preparation of QFY

*Ginseng Radix Et Rhizoma, Ziziphi Spinosae Semen*, and *Polygalae Radix* were purchased from Anguo Juyaotang Pharmaceutical Co. (Hebei, China). *Rehmanniae Radix Praeparata, Angelicae Sinensis Radix, Atractylodis Macrocephalae Rhizoma*, and *Glycyrrhizae Radix ET Rhizoma Praeparata Cum Melle* were purchased from Anxing Chinese Herbal Medicine Co. (Hebei, China).

The QFY sample solution was prepared in our laboratory using the above materials as raw materials and in accordance with the prescribed proportions (17). Dried pieces of *Ginseng Radix Et Rhizoma* (300 g), *Rehmanniae Radix Praeparata* (450 g), *Angelicae Sinensis Radix* (450 g), *Atractylodis Macrocephalae Rhizoma* (250 g), *Polygalae Radix* (250 g), *Glycyrrhizae Radix ET Rhizoma Praeparata Cum Melle* (150 g), and *Ziziphi Spinosae Semen* (crushed, 300 g) were immersed in 19,350 ml of distilled water for 30 min at room temperature and then decocted for 90 min. Subsequently, 15,050 ml of distilled water was added to the residue after filtering and decocting for 90 min. Next, extract solutions were mixed twice and concentrated to 2 g/ml.

### Animals

Transgenic mice with 5xFAD mutations [B6. Cg-Tg (APPSwFlLon, PSEN1*M146L*L286V) 6799Vas/Mmjax] (7) overexpress both mutant human APP (695) with the Swedish (K670N, M671L), Florida (I716V), and London (V717I) familial Alzheimer's disease (FAD) mutations and human PS1 harboring two FAD mutations, M146L and L286V, under transcriptional control of the neural-specific mouse Thy1 promoter (7). The 5xFAD mice were obtained from Jackson laboratories (Stock No. 034840, Bar Harbor, ME, USA) and bred to maintain the colony. Genotypes of animals were confirmed using PCR. Hemizygous 5xFAD mice and non-transgenic wild-type littermates were used. Both male and female 5xFAD mice were bred and raised to 2.5 months in the Experimental Center of Shandong University of Traditional Chinese Medicine. The mice were group-housed in a quiet facility and maintained in a 12-h light/dark cycle environment, with *ad libitum* access to food and water. Before the experiment, all mice were acclimatized to the experimental environment for 6 days. All animal-related experiments carried out have been reviewed and approved by the ethics committee of Shandong University of Traditional Chinese Medicine (Ethics No. SDUTCM20201228001). All efforts were taken to minimize the number of animals used and their suffering.

**Figure 1:**
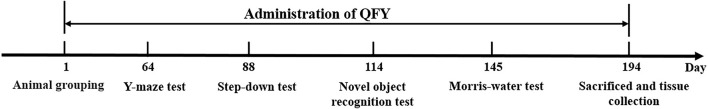
Schematic diagram of the experimental procedure.

The 2.5-month-old C57/B6XSJL and 5xFAD mice were randomly divided into six groups according to voluntary activity. Each group contained 9–13 mice: Group I: wild-type (WT) mice, Group II: 5xFAD transgenic mice, Group III: 5xFAD+Donepezil (1.3 mg/kg/d), Group IV: 5xFAD+QFY (2.8 g/kg/d), Group V: 5xFAD+QFY (5.6 g/kg/d), and Group VI: 5xFAD+QFY (11.2 g/kg/d). Group IV, Group V, and Group VI mice were administrated intragastric with QFY. Group III mice were administrated with donepezil. Group I and Group II mice were given equal volumes of distilled water, respectively. According to the experimental timelines (Figure 1), the drug administration (total 194 days) and behavioral tests were carried out. After the behavioral experiment, the whole brain, cortex, and plasma of each mouse were collected for soluble Aβ analysis, immunohistochemical experiment, and cytokine analysis.

### Behavioral tests

#### Y-maze test

The Y-maze test was performed to evaluate mouse short-term spatial memory (18–20). The Y-maze (XR-XY1032, 35 cm × 5 cm × 15 cm, Shanghai Xin Ruan Information Technology Co., Ltd*)* consists of three identical arms with different designs as visual markers for mice. The experimental process contains a 5 min-training stage and a 5 min-testing stage. During the first stage, the mice were only allowed to move in the start arm and another arm, with the novel arm (NA) blocked by a baffle. After 4 h, the NA was opened, and the mice were allowed to explore all three arms. The percentage of time spent in the NA, the number of entries into the NA, and the total distance traveled during the test were calculated using Tracking Master V3.0 Software (Shanghai VanBi Intelligent Technology Co., Ltd).

#### Step-down test

To measure the passive avoidance response, animals were used to conduct the step-down test (21, 22). The apparatus was a box with a platform (YLS-3TB, 4 cm × 3 cm × 2 cm, Jinan Yiyan Technology Development Co., Ltd) in the center of the grid floor. The floor consisted of parallel stainless steel strips. Before training, the mice were gently placed in the box to become accustomed to the environment for 3 min. If the mice get off the platform, they are given foot electric shock (60 ~ 80 V, 0.8 ~ 1.5 mA), and the mice jump onto the platform to avoid electric shock. This process lasts for 5 min. At the same time on the next day, mice were placed on the platform again, and the time to step down on the grid floor with all four paws for the first time (latency), and the number of errors were recorded using the Tracking Master V3.0 Software.

#### Novel object recognition test

The novel object recognition experiments were used to evaluate object recognition memory (23, 24). The mice were placed in a box (XR-XX117, 50 cm × 50 cm × 50 cm, Shanghai Xin Ruan Information Technology Co., Ltd) to acclimate for 10 min a day for 3 consecutive days. After 24 h of habituation, each mouse was allowed to explore for 5 min in an arena with two identical objects (objects A and B) placed at an equal distance. After 4 h, the mice were placed in the arena, but one of the objects was replaced with a new one (objects A and C). The next day, the mice were placed in the arena, but the object was replaced with another novel object again (objects A and D). The mice were again allowed to explore for 5 min. The time spent exploring each object was recorded using the Tracking Master V3.0 Software, and the preference index (PI) was calculated using the formula PI = TN/(TN + TF), where TN is the time spent exploring the novel object and TF is the time spent exploring of a familiar object.

#### Morris water maze test

The Morris water maze test was conducted to evaluate the spatial learning and memory ability of mice (25–27). The mice were placed in a circular pool with a diameter of 1.2 m (XR-XM101, Shanghai Xin Ruan Information Technology Co., Ltd.). This behavioral task included hidden-platform training (spatial learning) and probe trial (spatial memory) sessions. In the hidden-platform training session, the mice were allowed 4 daily trials in the presence of the platform for 5 subsequent days. In this session, mice were devoted into the pool facing the wall in one of the four quadrants. When the mice located the platform, it was allowed to stay on the platform for 10 s, and if the mice did not locate it within 60 s, it was placed on the platform for 10 s to familiarize the environment. The platform was removed after the training experiment, and the mice were placed from the water entry point in the diagonal quadrant where the platform was located and allowed to swim to search it for 60 s. During the whole MWM test, the escape latency (the time taken to find the hidden platform) in the hidden-platform training session, and the escape latency (the first time that the mice crossed the area of the removed platform), the number of crossing the target quadrant, and the time in the target quadrant in the probe trial session were recorded using the Tracking Master V3.0 Software.

#### Genotyping of transgenic mice

The genotyping method of transgenic mice as prescribed by Jackson Laboratories USA was used. DNA extraction was carried out using a DNA extraction kit (Accurate Biology, China, AG21010) following the manufacturer's protocol. The primers used in genotyping were Common-F, 5′-ACCCCCATGTCAGAGTTCCT-3′; Mutant-R, 5′-CGGGCCTCTTCGCTATTA C-3′; and Wild type-R, 5′-TATACAACCTTGGGGGATGG-3′. PCR products were identified through gel electrophoresis; amplified products were visualized using the Tanon 4800 chemiluminescence gel imaging system.

#### ELISA

The concentration of Aβ_1 − 42_ and Aβ_1 − 40_ in the plasma and cortex of the mice was measured using a precoated ELISA kit (Invitrogen, USA; KHB3441 and KHB3481, respectively) according to the manufacturer‘s instructions. The absorbance was measured at 450 nm using a full wavelength microplate reader (Thermo Fisher Scientific, MT90126728).

The level of CCL11 in the plasma and cortex of the mice was measured using a precoated ELISA kit (MULTISCIENCES, China, Cat# EK2030/224) according to the manufacturer‘s instructions. The absorbance was measured at 450 nm with a reference wavelength of 570 nm using a full wavelength microplate reader (Thermo Fisher Scientific, MT90126728).

#### Immunohistochemistry

Brain sections were dewaxed with xylene and ethanol, washed with distilled water, and incubated in a citrate buffer solution (pH 6.0) for antigen retrieval. Then, the endogenous peroxidase blocking solution was added for 10 min at room temperature (Sparkjade, China; Cat# EE0007). For histochemistry, rabbit monoclonal anti-Aβ_1 − 42_ was incubated (1:1,000, Abcam; Cat# ab201061) at 4°C overnight. After that, the reaction enhancement solution and enhance enzyme-labeled goat anti-rabbit IgG polymer (ZSGB-BIO, China; Cat# PV-9001) was incubated at room temperature for 20 min, respectively. After rinsing, the DAB kit (SparkJade, China; Cat# EE0017) coloration was performed for 2 min, and hematoxylin staining was done for 2 min to show the nucleus. Finally, the sections were sealed with neutral gum.

#### Multiplex bead analysis

Plasma samples were analyzed using a multiplex bead analysis. C-C motif chemokine ligand 2 (CCL2), C-C motif chemokine ligand 4 (CCL4), C-C motif chemokine ligand 5 (CCL5), granulocyte colony-stimulating factor (G-CSF), granulocyte-macrophage colony-stimulating factor (GM-CSF), interferon-γ (IFN-γ), interleukin-1β (IL-1β), interleukin-2 (IL-2), interleukin-4 (IL-4), interleukin-5 (IL-5), interleukin-6 (IL-6), interleukin-10 (IL-10), interleukin-17 (IL-17), and tumor necrosis factor α (TNF-α) were measured according to the manufacturer‘s instructions. Briefly, the plasma samples were incubated with premixed beads overnight at 4°C, and washed beads were further incurred with detection antibody for 1 h at room temperature followed by incubation with streptavidin–phycoerythrin for 30 min at room temperature. The samples were analyzed by using Luminex FLEXMAP 3D (Luminex, USA). The levels of CCL2?, CCL4?, CCL5, G-CSF, GM-CSF, IFN-γ, IL-1β, IL-2, IL-4, IL-5, IL-6, IL-10, IL-17, and TNF-α were detected using a Mouse Premixed Multi-Analyte Kit (RD Systems Inc, USA; Cat# LXSAMSA-15).

#### Statistical analysis

All data were expressed as mean ± SEM. GraphPad Prism 8.0 was used to plot and analyze data. Data between the two groups were compared using Student's *t*-test. Comparisons of data from multiple groups against one group were analyzed using a one-way analysis of variance (ANOVA) followed by Dunnett's *post hoc* test. A *P*-value of < 0.05 was taken as statistically significant.

## Results

### QFY improved cognitive impairments of the 5xFAD mice

In this study, the Y-maze test, step-down test, novel object recognition test, and Morris water maze test were performed to evaluate the effects of QFY on the cognitive function of the 5xFAD mice.

**Figure 2:**
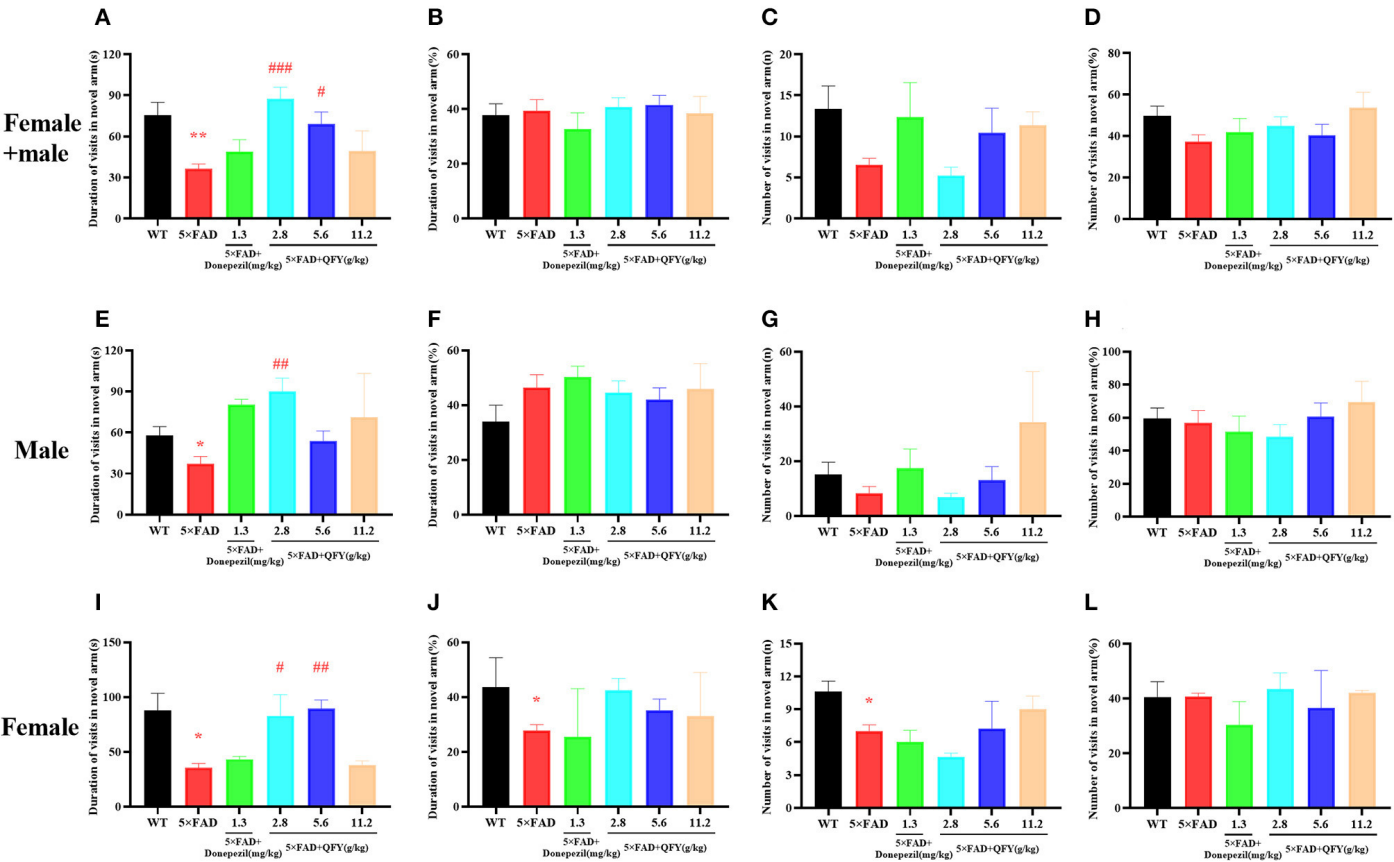
Effects of QFY on the ability of short-term spatial learning and memory in the 5xFAD mice. **(A, E, I)** Duration of visits in the novel arm. **(B, F, J)** Duration of visits in the novel arm (%). **(C, G, K)** Number of visits in the novel arm. **(D, H, L)** Number of visits in the novel arm (%). The values are mean ± SEM; *n* = 3–13. *P < 0.05, **P < 0.01 vs. WT mouse group by the unpaired Student's *t*-test; ^#^*P* < 0.05, ^##^*P* < 0.01, ^###^*P* < 0.01 vs. the 5xFAD mouse group by the one-way ANOVA followed by Dunnett's multiple comparisons test.

The Y-maze test was used to detect the short-term spatial learning and memory ability of the 5xFAD mice after the treatment of QFY. The results showed that the duration of visits in the novel arm of the 5xFAD mice was significantly shorter than the wild-type (WT) mice (Figure 2A, *P* < 0.01; Figures 2E, I, *P* < 0.05), the duration of visits in the novel arm (%) (Figure 2J, *P* < 0.05) were significantly decreased, and the number of visits in the novel arm were significantly reduced (Figure 2K, *P* < 0.05). Compared with the 5xFAD mice, the duration in the novel arm in the low-dose QFY (2.8 g/kg/d) (Figures 2A, E, *P* < 0.01; Figure 2I, *P* < 0.05) and medium dose (5.6 g/kg/d) (Figure 2A, *P* < 0.05; Figure 2I, *P* < 0.01) group was significantly prolonged. There was no significant difference in the duration of visits in the novel arm (%) (Figures 2B, F, J), the number of visits in the novel arm (Figures 2C, G, K) and the number of visits in the novel arm (%) (Figures 2D, H, L) between the QFY treatment groups and the 5xFAD group. There was no significant difference in all the indicators between male and female mice. These results suggest that the short-term spatial learning and memory ability of the 5xFAD mice was impaired, and donepezil has no effect on it. QFY can significantly improve the short-term spatial learning ability of the 5xFAD mice.

**Figure 3:**
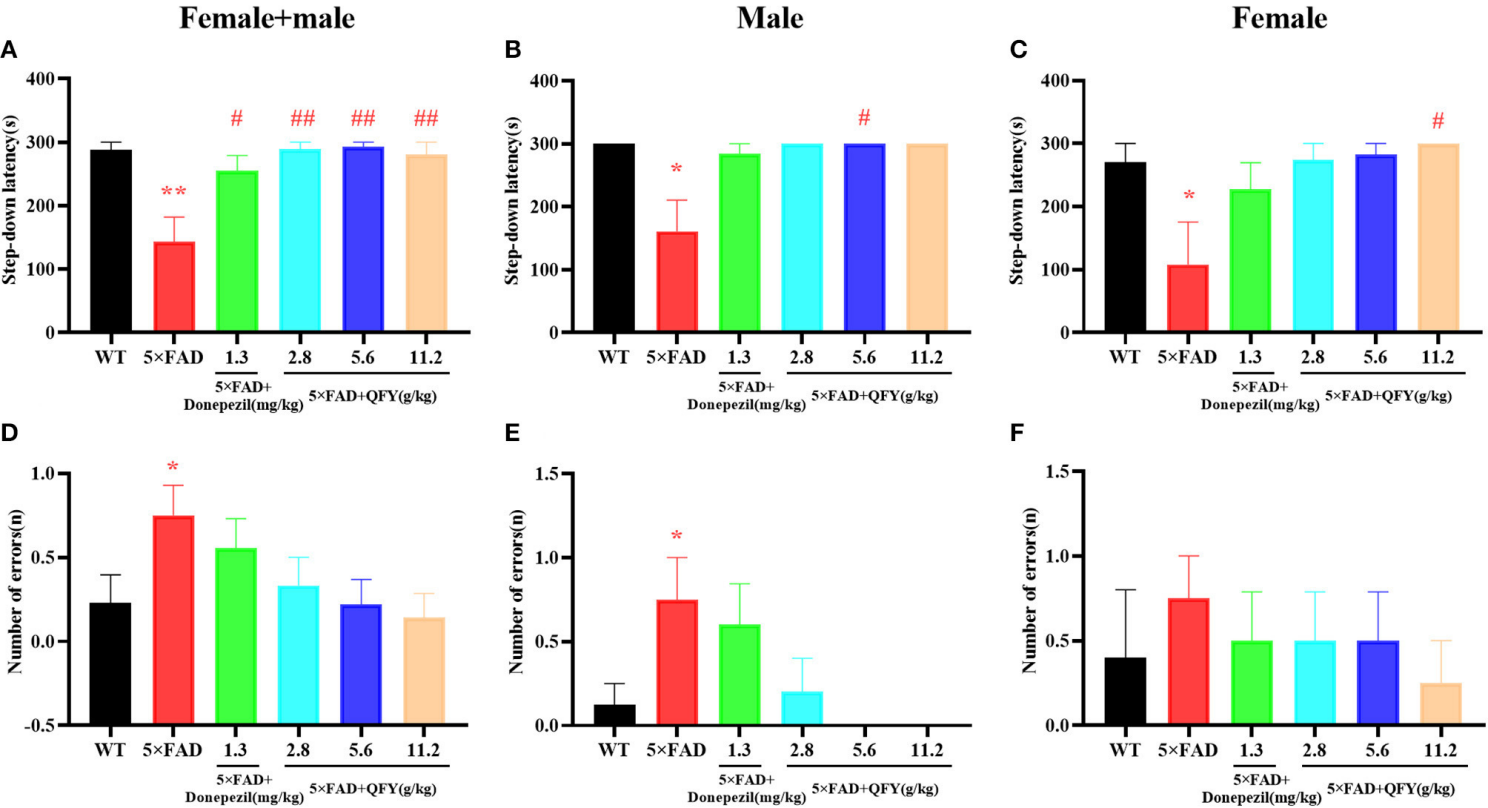
Effect of QFY on the passive avoidance ability in the 5xFAD mice. **(A–C)** Step-down latency. **(D–F)** Number of errors. The values are mean ± SEM; *n* = 3–13. *P < 0.05, **P < 0.01 vs. the WT mouse group by the unpaired Student's *t*-test; ^#^*P* < 0.05, ^##^*P* < 0.01 vs. the 5xFAD mouse group by the one-way ANOVA followed by Dunnett's multiple comparisons test.

After QFY treatment, the passive avoidance ability was detected by the step-down test (Figure 3). Compared with the WT group, the latency in 5xFAD mice significantly shortened (Figure 3A, *P* < 0.01; Figure 3B, *P* < 0.05; Figure 3C, *P* < 0.05), and the number of errors significantly increased (Figures 3D, E, *P* < 0.05). However, the administration of QFY significantly extended the latency (Figure 3A, *P* < 0.01; Figure 3B, *P* < 0.05; Figure 3C, *P* < 0.05). These data showed that the passive avoidance ability of 5xFAD mice was impaired, while the treatment of QFY and donepezil significantly improved it.

**Figure 4:**
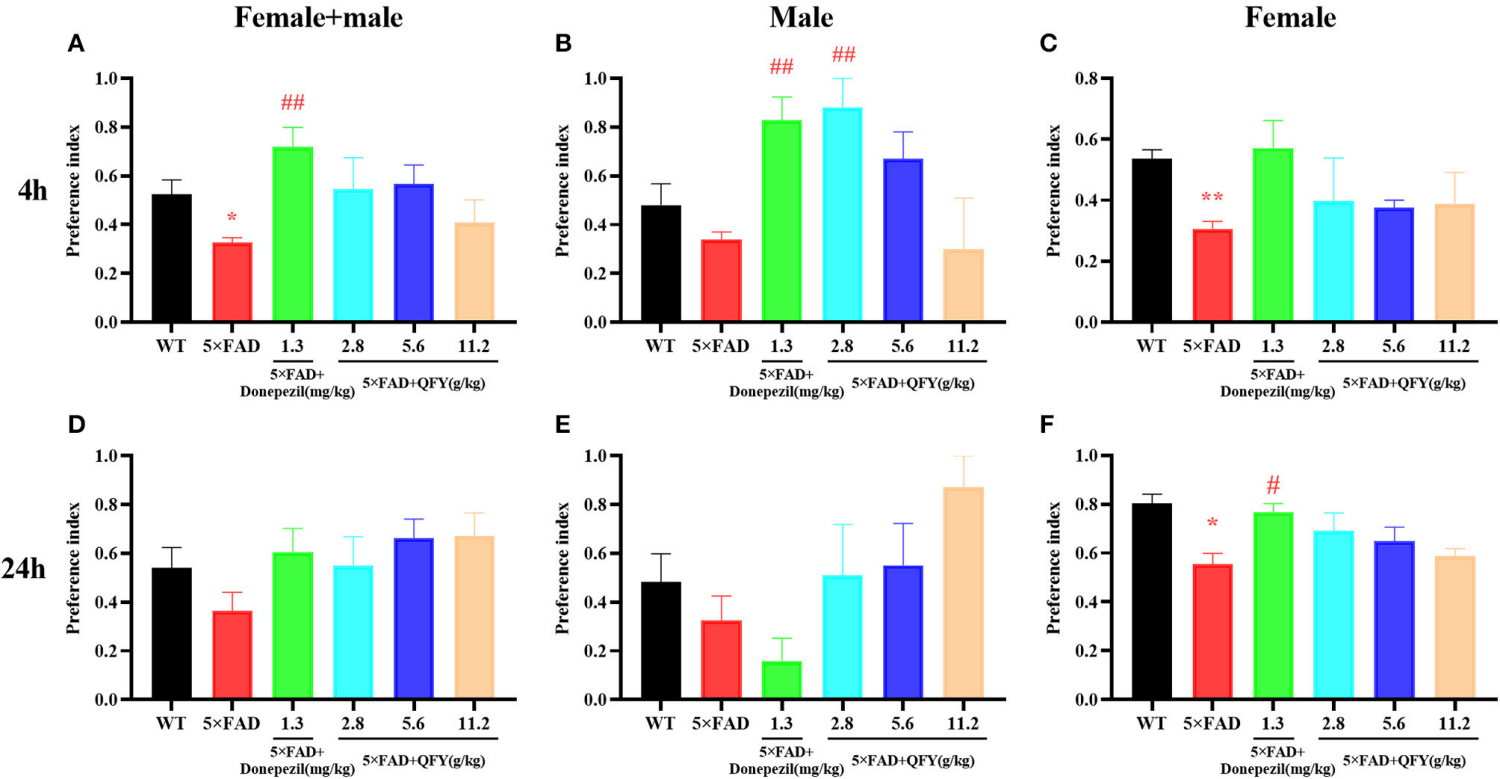
Effect of QFY on the object recognition memory in the 5xFAD mice. **(A–C)** 4h preference index. **(D–F)** 24 h preference index. The values are mean ± SEM; *n* = 3–13. *P < 0.05 vs. the WT mouse group by the unpaired Student's *t*-test; ^#^*P* < 0.05, ^##^*P* < 0.01, vs. the 5xFAD mouse group by the one-way ANOVA followed by Dunnett's multiple comparisons test.

The novel object recognition test was used to detect the object recognition memory ability of the mice with QFY treatment. The results of the present study showed that the preference index was significantly decreased in the 5xFAD mice compared with the WT group after 4 h of learning (Figure 4A, *P* < 0.05; Figure 4C, *P* < 0.01); Donepezil significantly increased the preference index of the 5xFAD mice after 4 h of learning (Figures 4A, B, *P* < 0.01), and 2.8 g/kg/d QFY significantly increased the preference index of the male 5xFAD mice after 4 h of learning (Figure 4B, *P* < 0.01). In addition, we found that the 24 h preference index of female 5xFAD mice decreased significantly (Figure 4F, *P* < 0.05), and donepezil improved it. There was no significant difference in the 24 h preference index (Figures 4D, E) between the QFY treatment groups and the 5xFAD group. These results indicate that deficits in the short-term object recognition memory of the 5xFAD mice were ameliorated by QFY and donepezil.

**Figure 5:**
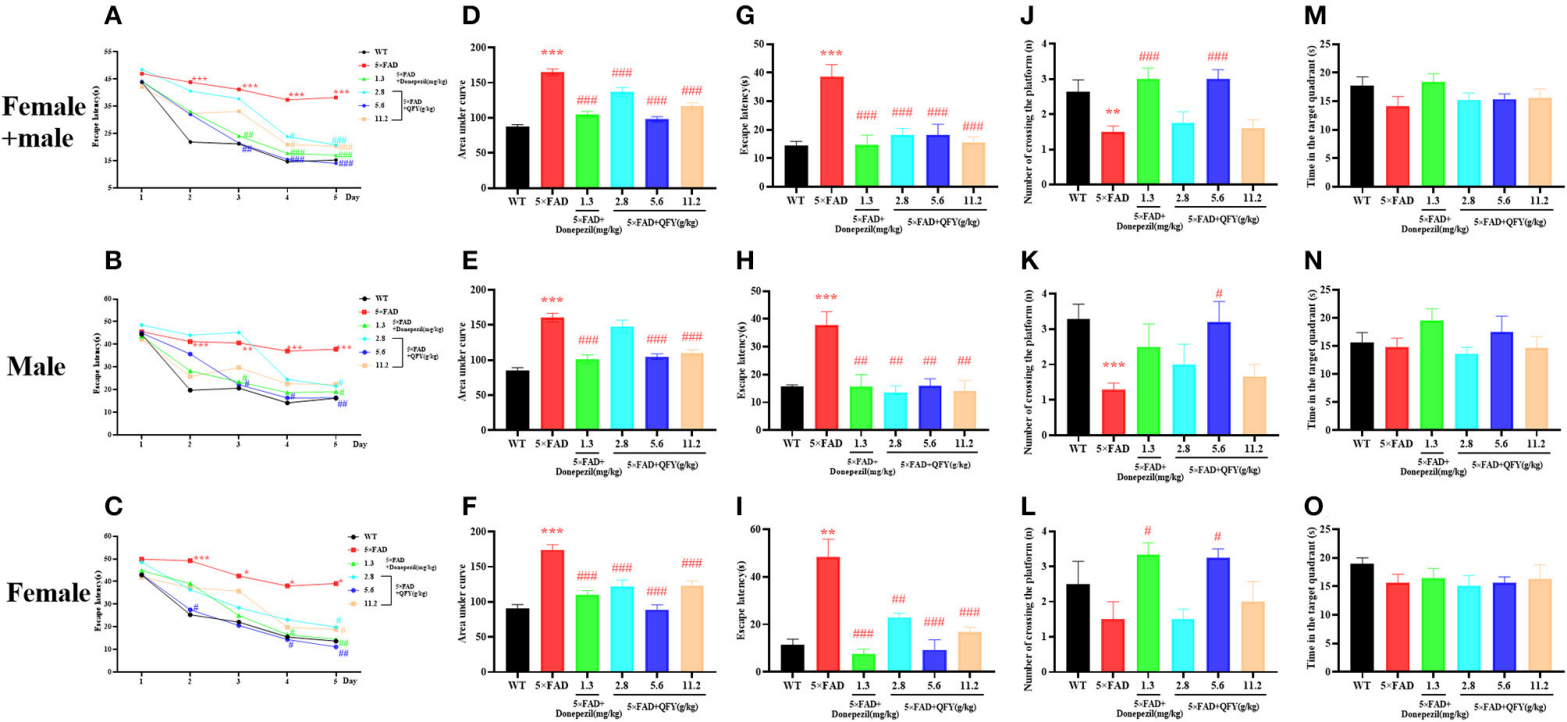
Effect of QFY on the ability of spatial learning and memory in the 5xFAD mice. **(A–C)** The escape latency of learning task. **(D–F)** Area under the curve of escape latency in the learning task. **(G–O)** The escape latency, number of crossing the platform, and time in the target quadrant of probe trial. The values are mean ± SEM; *n* = 3–13. **P < 0.01, ***P < 0.001 vs. the WT mouse group by the unpaired Student's *t*-test; ^#^*P* < 0.05, ^##^*P* < 0.01, ^###^*P* < 0.001 vs. the 5xFAD mouse group by the one-way ANOVA followed by Dunnett's multiple comparisons test.

The Morris water maze test was used to evaluate the spatial learning and memory ability of the mice after QFY treatment. In the learning task, the 5xFAD mice showed longer escape latency than the WT mice (Figures 5A–C, *P* < 0.01), and the area under the curve (AUC) of escape latency was significantly larger (Figures 5D–F, *P* < 0.01), and the 5xFAD mice of donepezil or QFY treatment had significantly shorter escape latency (Figures 5A–C, *P* < 0.01) and significantly smaller AUC (Figures 5D–F, *P* < 0.01) compared with the untreated 5xFAD mice. This data indicated that QFY (2.8, 5.6, and 11.2 g/kg/d) could ameliorate the impairment of spatial learning in the 5xFAD mice in the Morris water maze. During the probe testing, compared with the WT mice, escape latency was longer (Figures 5G–I, *P* < 0.01), and the number of crossing the platform was significantly reduced (Figures 5J, K, *P* < 0.01) in the 5xFAD mice. While the latency was decreased by donepezil (Figures 5G–I, *P* < 0.01) or QFY (2.8, 5.6 and 11.2 g/kg/d) (Figures 5G–I, *P* < 0.01) administration, the number of crossing the platform was increased significantly by donepezil (Figure 5J, *P* < 0.01; Figure 5L, *P* < 0.05) and 5.6 g/kg/d QFY (Figure 5J, *P* < 0.01; Figures 5K, L, *P* < 0.05) administration in the 5xFAD mice. There was no significant difference in time in the target quadrant (Figures 5M–O) between the groups. The results showed that the spatial learning and memory ability of the 5xFAD mice were impaired, and donepezil and QFY significantly improved this impairment.

### QFY treatment decreases Aβ_1 − 40_ and Aβ_1 − 42_ levels in the plasma and brain of the 5xFAD mice

**Figure 6:**
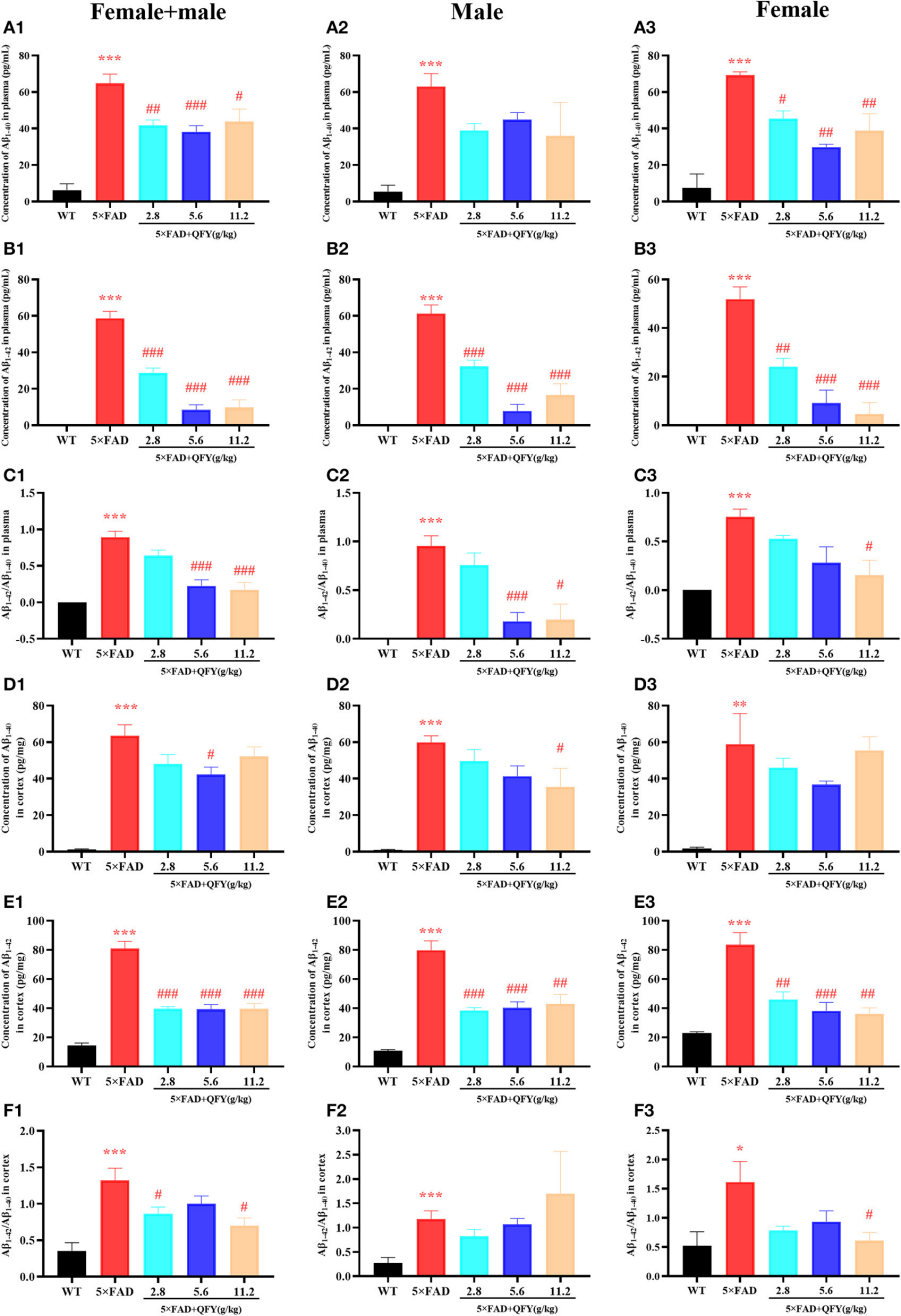
Effect of QFY on the Aβ level in the plasma and cortex of the 5xFAD mice. **(A1–C3)** The concentration of Aβ_1 − 40_, Aβ_1 − 42_, and Aβ_1 − 42_/Aβ_1 − 40_ in the plasma. **(D1–F3)** The concentration of Aβ_1 − 40_, Aβ_1 − 42_, and Aβ_1 − 42_/Aβ_1 − 40_ in the cortex. The values are mean ± SEM; *n* = 3–13. *P < 0.05, **P < 0.01, ***P < 0.001 vs. the WT mouse group by the unpaired Student's *t*-test; ^#^*P* < 0.05, ^##^*P* < 0.01, ^###^*P* < 0.001 vs. the 5xFAD mouse group by the one-way ANOVA followed by Dunnett's multiple comparisons test.

**Figure 7:**
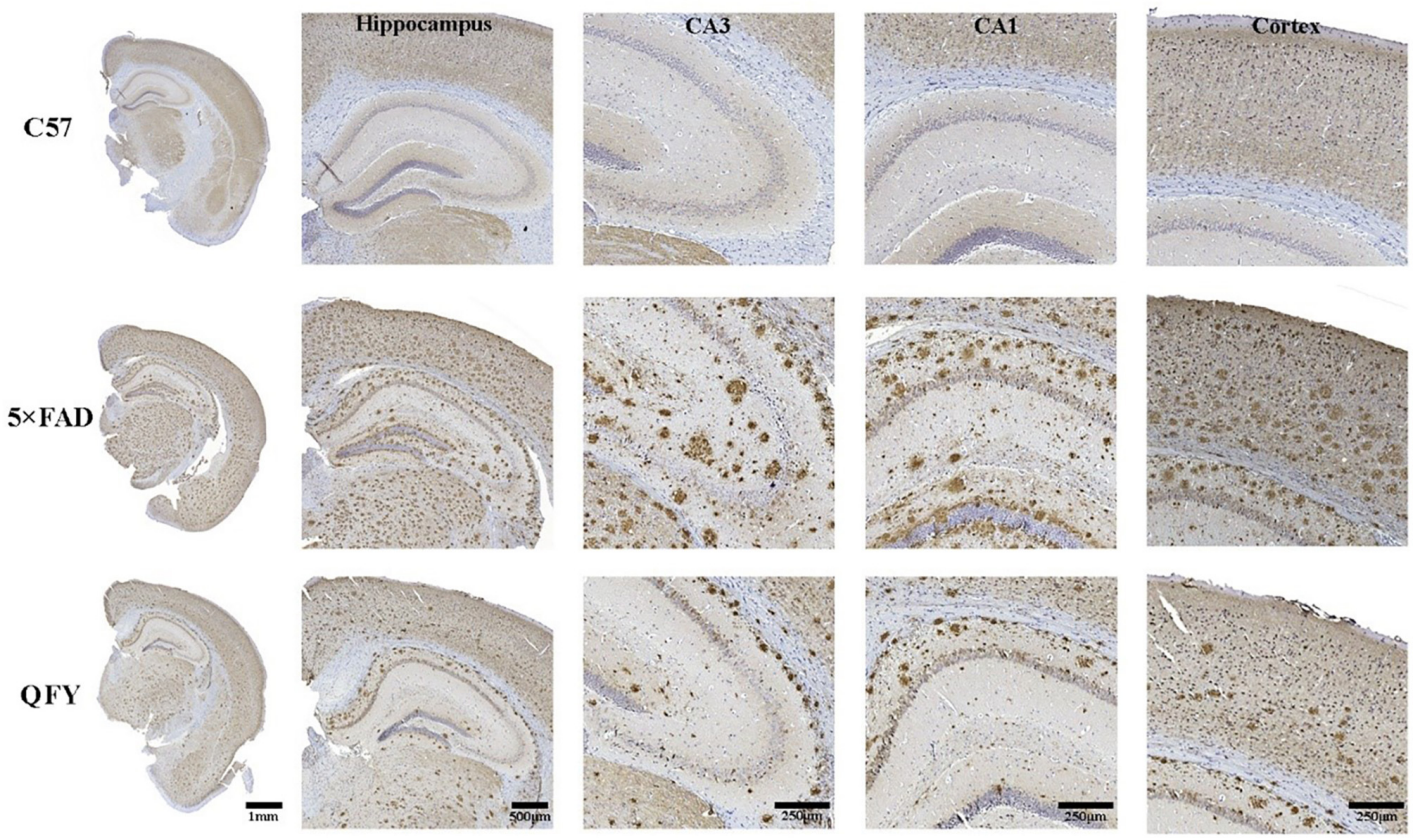
Effect of QFY on the Aβ deposits in the brain of the 5xFAD mice. Representative immunohistochemical images showing Aβ deposits in the brain, cortex, hippocampus, CA1, and CA3 of the WT and 5xFAD mice.

The results of the ELISA showed that the concentrations of Aβ_1 − 40_ and Aβ_1 − 42_ in the plasma (Figures 6A1–A3, B1–B3, *P* < 0.01) and cortex (Figures 6D1–D3, E1–E3, *P* < 0.01) of the 5xFAD mice were significantly higher than that of the WT mice. Treatment with QFY resulted in significantly lower levels of Aβ_1 − 40_ (Figures 6A1, A3, *P* < 0.05) and Aβ_1 − 42_ (Figures 6B1–B3, *P* < 0.01) in the plasma of the 5xFAD mice, and the levels of Aβ_1 − 40_ (Figures 6D1, D2, *P* < 0.05) and Aβ_1 − 42_ (Figures 6E1–E3, *P* < 0.01) in the cortex were reduced as well. In addition, we also found that the Aβ_1 − 42_/Aβ_1 − 40_ levels in the plasma (Figures 6C1–C3, *P* < 0.01) and cortex (Figures 6F1–F3, *P* < 0.01) of the 5xFAD mice were significantly increased, and the QFY could reverse this ration (Figures 6C1–C3, F1–F3, *P* < 0.05). The results of immunohistochemistry showed that numerous Aβ plaques were generated in the brains of the 5xFAD mice, whereas it was not observed in the WT mice (Figure 7). At the same time, QFY treatment significantly reduced the area of Aβ deposition in the whole brain, cortex, and hippocampus, such as CA1 and CA3 regions. These above findings indicate that Aβ deposition in the 5xFAD mice was alleviated after QFY administration.

### QFY treatment modulates abnormal cytokine production in the 5xFAD mice

**Figure 8:**
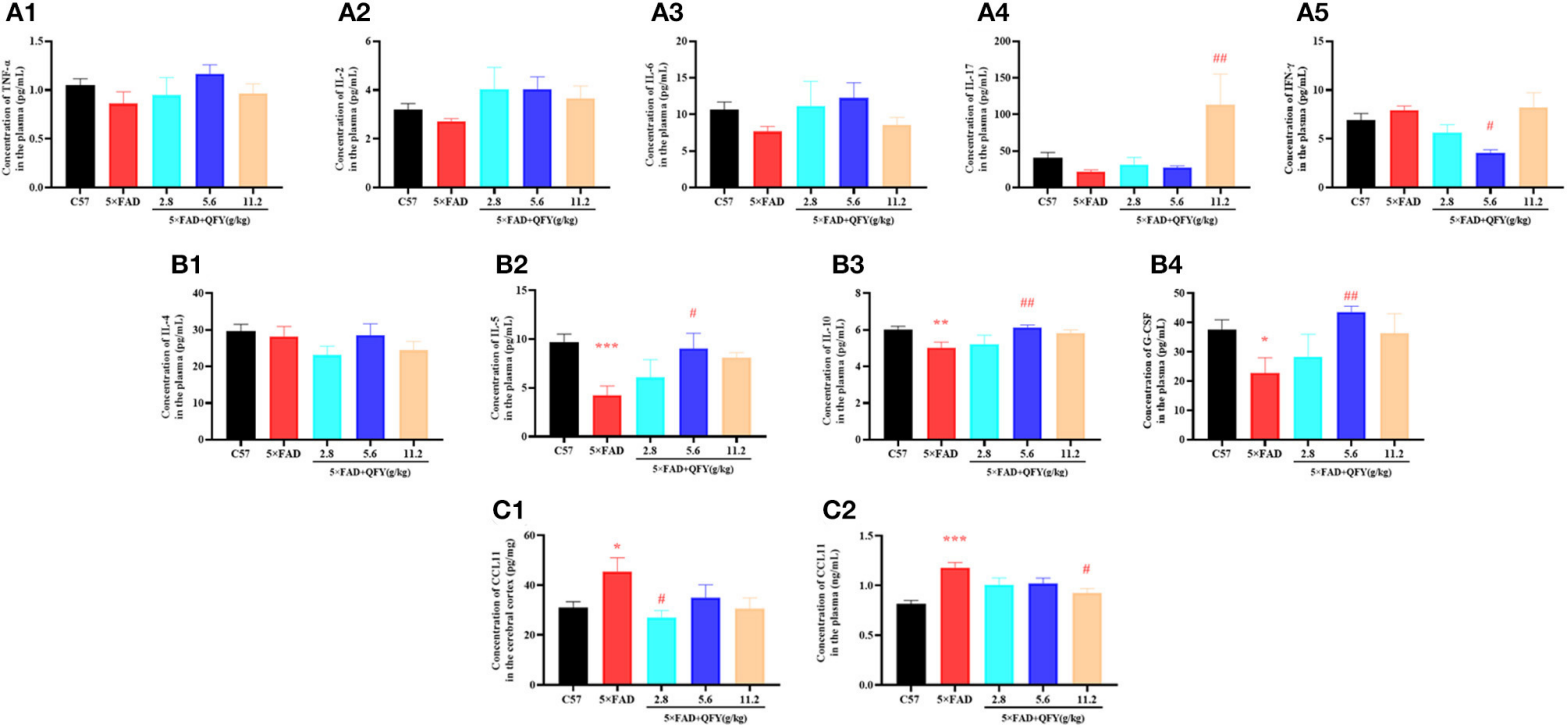
Effects of QFY on the cytokines in the 5xFAD mice. **(A1–B4)** The concentration of TNF-α, IL-2, IL-6, IL-17, IFN-γ, IL-4, IL-5, IL-10, and G-CSF in the plasma. **(C1, C2)** The concentration of CCL11 in the cortex and plasma. The values are mean ± SEM; *n* = 3–13. *P < 0.05, **P < 0.01, ***P < 0.001 vs. the WT mouse group by the unpaired Student's *t*-test; ^#^*P* < 0.05, ^##^*P* < 0.01 vs. the 5xFAD mouse group by the one-way ANOVA followed by Dunnett's multiple comparisons test.

To observe the effect of QFY on cytokine secretion, pro-inflammatory cytokines (TNF-α, IL-2, IL-6, IL-17, IFN-γ, and CCL11) and anti-inflammatory cytokines (IL-4, IL-5, IL-10, and G-CSF) in the plasma of the 5xFAD mice were detected using the multiplex bead analysis. We found that only the concentrations of anti-inflammatory factors IL-5 (Figure 8B2, *P* < 0.01), IL-10 (Figure 8B3, *P* < 0.01), and G-CSF (Figure 8B4, *P* < 0.05) were decreased in the plasma of the 5xFAD mice compared with WT. As expected, the levels of the three anti-inflammatory factors, IL-5 (Figure 8B2, *P* < 0.05), IL-10 (Figure 8B3, *P* < 0.01), and G-CSF (Figure 8B4, *P* < 0.01) were significantly restored after the administration of QFY (5.6 g/kg). For pro-inflammatory cytokines, the administration of QFY increased the level of IL-17 (Figure 8A4, *P* < 0.01) and decreased IFN-γ (Figure 8A5, *P* < 0.05) in the plasma of the 5xFAD mice. CCL11 is a famous cytokine promoting aging. The concentrations of CCL11 in the plasma and cortex were measured using an ELISA assay. The results showed that CCL11 concentrations were significantly increased in the cortex (Figure 8C1, *P* < 0.05) and plasma (Figure 8C2, *P* < 0.01) of the 5xFAD mice and significantly reduced by QFY treatment (Figures 8C1, C2, *P* < 0.05). There was no significant difference in the level of TNF-α, IL-2, IL-4 and IL-6 between the groups (Figures 8A1–A3, B1). This indicated that the cytokine secretion of the 5xFAD mice was abnormal, and QFY treatment regulated and restored this aberrant immune function in the 5xFAD mice.

## Discussion

**Figure 9:**
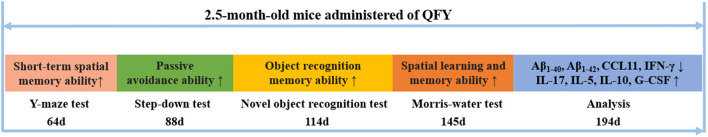
Qi-fu-yin attenuated cognitive disorders in the 5xFAD mice of Alzheimer's disease animal model by regulating immunity. ↑ represents an increase, ↓ represents a decrease.

AD is an age-related neurodegenerative disease with the pathological features of Aβ deposition. Mutations in the genes for APP, PS1, and PS2 increase the production of β-amyloid 42 (Aβ_1 − 42_) and cause AD (28). The accumulation of Aβ_1 − 42_ was observed in the 5xFAD mice at the age of 2 months. In addition, spatial learning and memory impairment was observed at 4 months old (7). The other several lines of evidence had shown that the 5xFAD mice exhibit impaired short-term spatial learning and memory ability (29), object recognition memory ability (24, 30, 31), avoidance response ability (32), and long-term spatial learning and memory ability (25, 27, 33, 34) compared with the WT mice, which is consistent with our results. The 5xFAD mice with long-term treatment of QFY showed significant better cognitive function in step-down test and Morris water maze test compared with 5xFAD mice. However, compared with the low and medium doses, a high dose of QFY did not improve the duration in the novel arm of the 5xFAD mice in the Y-maze test. In the Morris water maze test, the middle dose of QFY showed a better effect than the low dose of QFY; e.g., it showed better learning and memory ability on the third day of the probe testing and more times of number of crossing the platform. To sum up, all three doses of QFY improved the learning and memory ability of the 5xFAD mice, and the middle dose of QFY had the best effect. Taken together, this present study showed that the long-term administration of QFY improved cognitive impairment and alleviated Aβ deposition in the 5xFAD mice (Figure 9).

AD is a complex neurodegenerative disease, and its etiology is not caused by a single factor. A large number of studies have shown that senile plaques formed by the deposition of β-amyloid in the brain are one of the causes of AD (1, 35, 36). It has been found that neuroinflammation via microglia and astrocytes activated by Aβ plays an important role in the pathogenesis of AD (37). Cytokines are involved in complex abnormal cognitive processes in AD (38–40). Keena et al. (41) found that the levels of inflammatory cytokines and inflammatory markers in the blood of patients with AD and mild cognitive impairment are often high, and ~60% of late-onset and sporadic AD-related genes are related to inflammation. An epidemiological investigation found that middle-aged people with high levels of inflammation-related proteins in their blood have an increased risk of cognitive decline in the first few decades of old age, while middle-aged people who take anti-inflammatory drugs such as ibuprofen for a long time often have a lower risk of developing AD in their later years (42). There is a natural protective barrier in the central nervous system, i.e., the blood–brain barrier, which can effectively block external toxic substances from entering the nerve center and is of great significance for maintaining the homeostasis of the brain environment. However, long-term chronic peripheral inflammation can lead to the increase of brain capillary permeability and the destruction of blood–brain barrier and promote a large number of pathogens, immune cells, and their products to enter the brain to cause central inflammation, leading to AD (43).

The accumulation and deposition of Aβ in the brain is a critical step in the pathogenesis of AD (21, 44, 45). In our study, we detected that the long-term administration of QFY decreased the concentrations of Aβ_1 − 40_ and Aβ_1 − 42_ in the plasma and cortex of the 5xFAD mice. In addition, we also observed that LW-AFC significantly reduced Aβ deposition in the brain of the 5xFAD mice.

Increasing evidence suggests that neuroinflammation involves in the pathogenesis of AD along with classic pathological features such as misfolded and aggregated proteins, like Aβ and p-Tau (46). The increased levels of IL-1β, IL-6, and TNF-α were also found in the brain tissue of AD mice (47) and AD patients (48). On the contrary, anti-inflammatory factors, such as Il-4, IL-5, IL-10, and G-CSF, can inhibit central nervous system inflammation and have neuroprotective effects. Studies have shown that IL-4 accelerates the clearance of microglia by promoting CD36 expression and amyloid β-degrading enzymes (49). IL-10 can interact with glial cell surface receptors (IL-10Rs) to limit inflammation (50). IL-5 (51) and G-CSF (52) are also important anti-inflammatory cytokines and may be involved in regulating the progression of AD by inhibiting the effects of pro-inflammatory cytokines.

It is found that IL-17 is increased in APP/PS1 mice and Aβ-induced model mice and leads to neurotoxicity and cognitive decline through the IL-17/TRAF6/NF-κB pathway (53). However, the researchers found that the production of IL-17 in the 5xFAD mice decreased (54), and the level of IL-17 is also lower in AD patients (55), which is consistent with our research results. A study observed that IFN-γ levels in patients with mild and severe AD were higher than those in patients with moderate and MCI, respectively (56). Compared with WT, the IFN-γ level in 5xFAD mice was increased for the first time in this study. In *in vitro*, IL-5 prevented tau hyperphosphorylation and apoptosis induced by Aβ (25–35) through the JAK2 signaling pathway (57). G-CSF is a growth factor associated with AD improvement. It was found that the combination therapy of G-CSF and stromal cell-derived factor-1 reduced the deposition of Aβ and improved the apoptosis of Aβ-induced model rats (58). Similarly, we found for the first time that the level of IL-5 and G-CSF in the 5xFAD mice decreased significantly. In addition, the analysis of hippocampus mRNA expression showed that the level of anti-inflammatory cytokine IL-10 increased in the 5xFAD/C6-KO mice (59). Our results showed that QFY reduced the levels of pro-inflammatory cytokines IFN-γ, restored the level of IL-17, and increased the levels of anti-inflammatory cytokines such as IL-5, IL-10, and G-CSF.

Chemokines were originally described as factors that regulate the migration of peripheral immune cells, participate in inflammatory reactions, and can further lead to neurodegenerative diseases (60, 61). Notably, it was found that CCL11 and its receptor were involved in cognitive impairment in AD patients (62, 63) and AD model animals (64–66). A study shows that with the increase of age, the levels of CCL11 in plasma and cerebrospinal fluid of humans and mice increase, which will reduce synaptic plasticity, inhibit nerve regeneration, and lead to cognitive impairment (63). CCL11 is also called “endogenous cognitive deterioration chemokine” or “accelerating brain aging chemokine” (67). Bijay (68) found that CCL11 significantly promoted the migration of glial cells and induced glial cells to produce reactive oxygen species by upregulating nicotinamide adenine dinucleotide phosphate oxidase 1 (NOX1), which eventually led to neuronal death. In addition, the study shows that the expression of CCL11/CCR3 is very likely to be an important factor involved in the inflammatory induction and demyelination of AD (6). In this present study, the content of CCL11 in the plasma and brain of the 5xFAD mice was detected for the first time, and it was found that QFY reversed the excessive level of CCL11 in the brain and plasma of the 5xFAD mice.

Taken together, our data indicated that QFY improved object recognition, passive avoidance responses, and spatial learning and memory in the 5xFAD mice and reduced Aβ deposits. This improvement may be achieved by regulating abnormal immunity in the 5xFAD mice. Our findings support QFY as a potential therapeutic agent for AD.

## Data availability statement

The raw data supporting the conclusions of this article will be made available by the authors, without undue reservation.

## Ethics statement

All animal-related experiments carried out have been reviewed and approved by the Ethics Committee of Shandong University of Traditional Chinese Medicine (Ethics No. SDUTCM20201228001). All efforts were taken to minimize the number of animals used and their suffering.

## Author contributions

All authors listed have made a substantial, direct, and intellectual contribution to the work and approved it for publication.
